# Assessment of Moral Injury in Veterans and Active Duty Military Personnel With PTSD: A Review

**DOI:** 10.3389/fpsyt.2019.00443

**Published:** 2019-06-28

**Authors:** Harold G. Koenig, Nagy A. Youssef, Michelle Pearce

**Affiliations:** ^1^Duke University Medical Center, Durham, NC, United States; ^2^King Abdulaziz University, Jeddah, Saudi Arabia; ^3^Ningxia Medical University, Yinchuan, China; ^4^Medical College of Georgia, Augusta University, Charlie Norwood VA Medical Center, Augusta, GA, United States; ^5^Department of Family and Community Medicine, Center for Integrative Medicine, University of Maryland School of Medicine, Baltimore, MD, United States

**Keywords:** moral injury, internal conflict, posttraumatic stress disorder, Veterans, Active Duty Military, screening

## Abstract

**Background:** Moral injury (MI) involves distress over having transgressed or violated core moral boundaries, accompanied by feelings of guilt, shame, self-condemnation, loss of trust, loss of meaning, and spiritual struggles. MI is often found in Veterans and Active Duty Military personnel with posttraumatic stress disorder (PTSD). MI is widespread among those with PTSD symptoms, adversely affects mental health, and may increase risk of suicide; however, MI is often ignored and neglected by mental health professionals who focus their attention on PTSD only.

**Methods:** A review of the literature between 1980 and 2018 conducted in 2018 is presented here to identify scales used to assess MI. Databases used in this review were PsychInfo, PubMed (Medline), and Google Scholar. Search terms were “moral injury,” “measuring,” “screening,” “Veterans,” and “Active Duty Military.” Inclusion criteria were quantitative measurement of MI and health outcomes, Veteran or Active Duty Military status, and peer-review publication. Excluded were literature reviews, dissertations, book chapters, case reports, and qualitative studies.

**Results:** Of the 730 studies identified, most did not meet eligibility criteria, leaving 118 full text articles that were reviewed, of which 42 did not meet eligibility criteria. Of the remaining 76 studies, 34 were duplicates leaving 42 studies, most published in 2013 or later. Of 22 studies that assessed MI, five used scales assessing multiple dimensions, and 17 assessed only one or two aspects (e.g., guilt, shame, or forgiveness). The remaining 20 studies used one of the scales reported in the first 22. Of the five scales assessing multiple dimensions of MI, two assess both morally injurious events and symptoms and the remaining three assess symptoms only. All studies were cross-sectional, except three that tested interventions.

**Conclusions:** MI in the military setting is widespread and associated with PTSD symptom severity, anxiety, depression, and risk of suicide in current or former military personnel. Numerous measures exist to assess various dimensions of MI, including five multidimensional scales, although future research is needed to identify cutoff scores and clinically significant change scores. Three multidimensional measures assess MI symptoms alone (not events) and may be useful for determining if treatments directed at MI may both reduce symptoms and impact other mental health outcomes including PTSD.

## Introduction

### Rationale

Experiences during combat have long been known to cause internal moral or ethical conflicts (1). “Moral injury” (MI) has become the term used to describe the moral suffering that results from experiences involving violence against others during the course of police work or during wartime (2, 3). There are many definitions of MI in the literature (see Hodgson & Carey for a sense of the diversity of such definitions) (4). For example, MI acquired during combat has been described as “a deep sense of transgression including feelings of shame, grief, meaninglessness, and remorse from having violated core moral beliefs” (p xiv, Brock & Lettini) (5), including “a betrayal of what’s right, by someone who holds legitimate authority, in a high-stakes situation” (Shay, p 183) (6). Such feelings relate to what one has done (killed combatants or innocents, dismembered bodies, maltreated others, or deserted comrades during battle), what one has failed to do (protected innocents or prevented the death of fellow soldiers), or what one has observed others do or fail to do. MI may also involve intense feelings of betrayal by those in authority, either in or outside of the military, and may for some include religious or spiritual struggles and even a complete loss of religious faith (7) resulting from experiences during wartime.

MI has been distinguished from posttraumatic stress disorder (PTSD), which may occur alongside it (5, 8, 9). MI is considered a syndrome separate and distinct from PTSD, although with some definitional overlap between the two (particularly in the affective domain, i.e., PTSD symptom cluster D) [*Diagnostic and Statistical Manual of Mental Disorders*, 5th edition (DSM-5) (10)]. One can have PTSD without MI, MI without PTSD, or both together. According to DSM-5, the diagnosis of PTSD is based on the exposure to a severe traumatic stressor (Criterion A) and the presence of four major fear and trauma-based symptom clusters that cause problems in daily functioning: intrusive nightmares and flashbacks (Criterion B), avoidance (Criterion C), emotional negativity and numbing (Criterion D), and hyperarousal and irritability (Criterion E). In contrast, MI results from transgressions committed, observed, or learned about that conflict with moral beliefs (11) and is a syndrome characterized by guilt, shame, feelings of betrayal, difficulty forgiving, loss of meaning, loss of trust, self-condemnation, spiritual struggles, and feelings of inner conflict over the moral implications of those transgressions (3–7, 12–14). Experiences during war may be severely traumatic (as in Criterion A for the diagnosis of PTSD), morally injurious, or both. For some individuals, transgressing cherished moral values or experiencing betrayal by trusted others in high stakes situations may be severely traumatic, whereas for others, these events may be very distressing yet not reach the threshold for PTSD (i.e., Criterion A, involving exposure to death, threatened death, actual or threatened serious injury, actual or threatened sexual violence, and Criteria B-E in DSM-5). A MIE (morally injurious event), like any distressing event that has occurred in the past, cannot be changed; however, the symptoms that result from these events and continue to cause distress and dysfunction may be assessed and treated.

One reason that MI has received increasing attention over the past decade is the possibility that it may block successful treatment of PTSD, one of the most common mental disorders in Veterans and Active Duty Military (ADM) (15, 16) that is often resistant to both pharmacological and psychological therapies (17, 18). The identification and treatment of MI among those with PTSD may be key to the management and ultimate resolution of the latter (6, 10). MI is recognized as one of the five stress outcomes noted in the *Consensus Recommendation for Common Data Elements for Operational Stress Research and Surveillance* report by U.S. Armed Forces and Veterans Administration (VA) experts, and “case identification” is one of seven components of the mental health intervention spectrum noted in that report (19).

Systematic research has shown that MI is common among Veterans with PTSD symptoms. One study reported at least one MI symptom of significant severity in over 90% of 373 Veterans (59% with five or more such symptoms) (20) and in over 80% of 103 ADM (52% with four or more symptoms) (21). The seriousness of MI has been underscored by its association in Veterans with a host of adverse mental health outcomes, including PTSD (12, 22, 23), depression and anxiety (21, 23–26), and increased risk of suicide (27–29). Several of these studies show that MI is associated with depression, anxiety, and suicide, even after controlling for severity of PTSD symptoms (12, 19, 27–29), further justifying MI as a syndrome separate from PTSD. However, there is no measure of MI that uses gold standard methodology here, underscoring the importance of understanding what measures are available for current use and how understanding these may help inform the development of more robust measures. While MI in military settings has been discussed since the early 1980s, systematic research providing an evidence base on the topic has been only relatively recent. As a result, many mental health professionals may not have even heard of MI, and the condition can often go unrecognized and ignored when the clinician’s primary focus is on PTSD.

### Research Question

The purpose of this study was to review measures used to assess MI that clinicians may use for screening and behavioral health investigators for conducting research in current and former military personnel. This review focused on scales that assess single or only a few dimensions of MI (guilt, shame, difficulty forgiving, loss of meaning, moral objections, and transgressions) and those that more comprehensively assess multiple aspects of this construct. In order to be comprehensive, we have included measures that address only one or two aspects of MI (e.g., transgressions, guilt, and shame). However, we do not believe that those measures are assessing the construct of MI as a unique phenomenon, but only assess certain dimensions of MI and are therefore incomplete in themselves.

Measures are distinguished in terms of whether they assess morally injurious events (experiences in war that cannot be changed) or MI symptoms (feelings about those events that can be altered by therapeutic interventions), or both events and symptoms. Reviewed are studies using these scales for the first time to assess MI in Veterans (including original validation studies) and later studies that have used those scales in military populations. Based on this review, recommendations are made on the best measures to use depending on the clinician’s or researcher’s goal. Treatments for MI are also briefly discussed.

## Method

### Study Design

The review focused on studies that developed or used measures of MI to examine health outcomes in present and former military personnel. Because the emphasis was on “moral injury,” this term was included either alone or with the keywords “Active Duty Military,” “Veterans,” “measuring,” and “screening.” The Boolean operators “and”/“or” were used between search terms to reduce the number of articles to those meeting the inclusion and exclusion criteria for this review. Inclusion criteria were 1) quantitative measurement of MI (scales including more than one item), 2) assessment of Veterans or ADM, 3) quantitative measurement of health outcomes, and 4) publication in a peer-reviewed academic journal in the English language. Excluded were literature reviews, dissertations, book chapters, letters to the editor, case reports, and qualitative studies.

### Search Strategy

**Figure 1:**
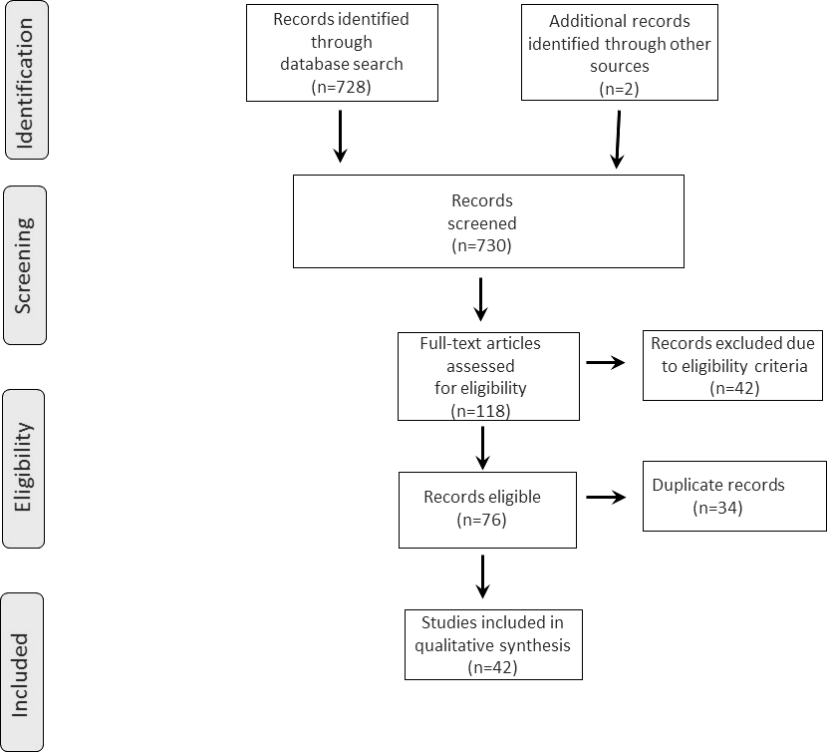
Flow diagram of selection of studies (PRISMA chart).

The search strategy involved four stages. The first stage involved a search of the literature between 1980 and April 3, 2018, using the databases PubMed, PsychInfo, and Google Scholar. Second, the titles of promising articles were reviewed to identify studies that appeared to meet the inclusion criteria. Third, abstracts of these articles were reviewed. Finally, the full texts of articles that passed the initial screens were retrieved and examined more closely to ensure that inclusion and exclusion criteria were met. Each of the three co-authors independently conducted the review, screened relevant articles, and then agreed by consensus on the articles that met the criteria above. **Figure 1** provides a Preferred Reporting Items for Systematic Reviews and Meta-Analyses (PRISMA) chart describing how studies were selected for this review.

## Results

The search term “moral injury” alone identified 62 articles in PubMed and 160 articles in PsychInfo, which represented the total number of articles identified by the three reviewers (all reviews were independently conducted in March and early April 2018). Given the number of articles in those two databases were relatively few, all were screened. When the keyword “moral injury” was used to search the Google Scholar database, however, over 5,000 articles were retrieved. To narrow down the search based on study inclusion criteria, the terms “Veterans,” “Active Duty Military,” “measuring,” and “screening” were added to the search term “moral injury” reducing the number of articles to 446, all of which were screened. Thus, search of the three databases identified 728 possible studies. Two additional studies were identified (known by the authors to be published soon), increasing the total to 730. Of those, 118 looked promising enough to download the full text articles and review them more carefully for inclusion criteria. Of those, 42 were eliminated for failing to meet inclusion and exclusion criteria leaving 76 eligible records. After excluding 34 duplicates, this resulted in the final 42 studies for this review. Most of these (93%) were published in 2013 or later, and 78% were published in 2017 or 2018, underscoring the recent attention paid to this topic.

Of the 42 studies, 17 studies developed or used previously published measures that assessed only one or two aspects of MI (e.g., guilt, shame, or forgiveness), and five studies reported the development of scales that assessed multiple dimensions of MI (**Table 1**). In addition, 20 studies used a scale reported in one of the first 22 studies published earlier; these were included to provide a sense of the scales most commonly used today by researchers to measure MI (**Table 2**). Except for one randomized clinical trial (RCT), one non-randomized trial, and one planned RCT, studies were all cross-sectional in design. No study established a cutoff to indicate significant symptom levels on a scale requiring clinical attention, nor did any study report clinically significant change scores for a scale. Now reviewed are the studies describing the 22 scales identified in this review.

**Table 1:** Characteristics of studies developing or using scales to assess moral injury (ordered by year of publication) (n = 22).

Reference (abbreviation)	Design	Population Studied	Events vs. Symptoms	Moral Injury Dimension	No. Items (Rating)	Source Scale	Psychometrics
Henning and Frueh (30) (CGS)	CS	40 Veterans with PTSD	Symptoms only	Guilt	15 (1 vs. 0)	Authors	α = .78
Stein et al. (31)	CS	122 ADM	Event Categories	MI by self MI by others	2 (1 vs. 0)	Authors	kappa = .74-.90
Gray et al. (32)	NRCT	44 Marines	Cognitions and beliefs	Trauma-related cognitions	33 (1-7)	Posttraumatic Cognitions Inventory	—
Nash et al. (12)	CS	533 Marines 503 Marines	Events and symptoms	Transgressions by self, others, and betrayal	9 (1-6)	Authors	2 factors (F) F1 α = .89 F2 α = .82
Bryan et al. (27)	CS	69 ADM	Symptoms only	Guilt	6 (0-4)	Personal	α = .85
				Shame	10 (0-4)	Feelings Questionnaire (PFQ-2)	α = .86
Ritov et al. (33)	CS	147 ADM (Israeli)	Symptoms (moral response to events)	Moral objections	4 (1-7)	Authors	α = .83
Currier et al. (34) (MIQ-M)	CS	131 Veterans 82 Veterans	Events and symptoms	Betrayal, moral violations, guilt, others	19 (1-4)	Authors	1 factor α not reported
Bryan et al. (29)	CS	474 ADM or Veterans	Symptoms only	Self-forgiveness	6 (1-7)	Heartland Forgiveness Scale	α = .84
Hijazi et al. (35)	CS	167 Veterans	Symptoms only	Wrongdoing	5 (0-4)	Trauma-Related Guilt Inventory (TRGI) subscale	α = .78
Crocker et al. (36)	CS	127 Veterans	Symptoms only	Shame	24 (0-4)	Internalized Shame Scale;	α = .96
				Guilt	32 (0-4)	TRGI	α = .87-.91
Campbell (37) (M-CoSS)	CS	378 Sailors 27 ADM	Symptoms only	Maladaptive shame regulation	6 by 4	Author	α = .89
Yan (38)	CS	100 Veterans	Events only	Combat experiences (aftermath of battle)	30 (1 vs. 0)	Deployment Risk & Resilience Inventory (DRRI)	α = .85-.86
Dennis et al. (39)	CS	603 Veterans	Events and symptoms	Atrocities committed Guilt (global)	6 (1-5) 4 (0-4)	Vietnam Stress Invent. TRGI subscale	α = .87 α = .88
Frankfurt et al. (40)	CS	190 Veterans	Events and symptoms	Transgressive acts Feeling guilty	8 (1 vs. 0) 1 (0-5)	Clinician Administered PTSD Scale-IV	K = .72
Lancaster (41)	CS	161 Veterans	Events and symptoms	Transgressions/betrayal Transgressive acts Shame and guilt	6 (1-6) 7 (1 vs. 0) 10 (1-5)	MIES (partial) Author State Shame and Guilt Scale	— — α = .90 shame α = .88 guilt
Maguen et al. (42)	RCT	33 Veterans with PTSD	Symptoms and beliefs	Maladaptive beliefs about killing	55 (1-5)	Author (Killing Cognitions Scale)	—
Currier et al. (26) (EMIS-M)	CS	286 Veterans 624 Veterans	Symptoms only	Self-directed, Other-directed (shame, guilt, betrayal, etc.)	17 (1-5)	Authors	2 factors α = .94-.95 (total) Test-retest α = .80
Koenig et al. (23) (MISS-M-LF)	CS	214 Veterans 213 Veterans (with PTSD symptoms)	Symptoms only	Guilt, shame, moral concerns, betrayal, religious struggles, loss of faith, loss of meaning, loss of trust, difficulty forgiving, self-condemnation	45 (1-10)	Items from multiple established scales, and study authors	1-2 factors per subscale Overall α = .92 Test-retest α = .91
Koenig et al. (24) (MISS-M-SF)	CS	214 Veterans 213 Veterans (as above)	Symptoms only	Same as above MISS-M-LF	10 (1-10)	Based on MISS-M-LF	1 item/scale Overall α = .73 Test-retest α = .87
Nazarov et al. (43) (DEX)	CS	4854 ADM (Canadian)	Events only	Potential moral injury events (PMIEs)	3 (1 vs. 0)	US/Canada Combat Experiences Scale	None reported
Bryan et al. (44)	CS	930 ADM	Symptoms only	Anger outward, hostility inward, shame, guilt, sorrow;	15 (1-5)	Differential Emotions Scale-IV	α = .85-.93
				low cohesion	5 (1-5)	DRRI-2	α = .91
Currier et al. (45)	CS	1124 Veterans	Symptoms only	Religious/spiritual struggles	22 (1-5)	Religious and Spiritual Struggles Scale	α = or >.90

CS, cross-sectional; ADM, Active Duty Military; *α*, Cronbach’s alpha (internal reliability).

**Table 2:** Other studies in which moral injury scales in **Table 1** were used (ordered by year of publication) (n = 20).

Reference	Design	Population Studied	Events vs. Symptoms	MI Dimension	No. Items (Rating)	Source Scale	Psychometrics
Bryan et al. (46)	CS	97 ADM	Symptoms only	Guilt	6 (0-4)	PFQ-2	α = .85
Bryan et al. (28)	CS	151 ADM	Events and symptoms	Transgressions by self, by others, and betrayal	9 (1-6)	MIES	3 factors reported α’s > .79 reported
Currier et al. (47)	CS	131 Veterans	Events and symptoms	Betrayal, moral violations, guilt, others	19 (1-4)	MIQ-M	—
Bryan et al. (48)	CS	464 ADM or Veterans	Symptoms only	Guilt	6 (0-4)	PFQ-2	α = .85
Bryan et al. (22)	CS	151 ADM 935 ADM	Events and symptoms	Transgressions by self, by others, and betrayal)	9 (1-6)	MIES	3 factors demonstrated α’s = .83-.96
Wisco et al. (49)	CS	564 Veterans	Events and symptoms	Transgressions by self, by others, and betrayal	9 (1-6)	MIES	3 factors reported α = .88 (total)
Lancaster and Erbes (50)	CS	182 Veterans	Symptoms only	Shame	10 (0-4)	PFQ-2	α = .92
				Guilt	5 (0-4)		α = .88
Ferrell et al. (51)	CS	37 Veterans	Events and symptoms	Betrayal, moral violations, guilt, others	19 (1-4)	MIQ-M	—
Currier et al. (52)	CS	125 Veterans	Events and symptoms	Betrayal, moral violations, guilt, others	19 (1-4)	MIQ-M	—
Evans et al. (25)	CS	155 Veterans	Events and symptoms	Transgressions, by self, by others, and betrayal	9 (1-6)	MIES	3 factors reported α = .91
Houtsma et al. (53)	CS	522 ADM	Events and symptoms	Transgressions by self, by others, and betrayal	9 (1-6)	MIES	3 factors reported α’s = .75-.94
Jordan et al. (54)	CS	867 Marines	Events and symptoms	Transgressions by self and betrayal	7 (1-6)	MIES (partial)	2 factors reported α’s = .84-.93
Martin et al. (55)	CS	562 ADM	Symptoms only	Betrayal	3 (1-6)	MIES (partial)	1 factor reported α = .86
Cunningham et al. (56)	CS	988 Veterans with PTSD	Symptoms only	Guilt (hindsight bias, wrongdoing, lack of justification)	22 (0-4)	TRGI cognitions	α = .91
Yeterian et al. (planned) (57)	RCT	186 Veterans	Symptoms only	Guilt Shame	32 (0-4) 24 (0-3)	TRGI TRSI	— —
Dedert et al. (58)	CS	50 Veterans	Symptoms only	Guilt (hindsight bias, wrongdoing, lack of justification)	18 (0-4)	TRGI cognitive subscales	—
Volk and Koenig (21)	CS	103 ADM w PTSD symptoms	Symptoms only	10 MI symptom categories	45 (1-10)	MISS-M-LF	α = .92
Norman et al. (59)	CS	254 ADM	Symptoms only	Guilt (hindsight, bias, wrongdoing, lack of justification)	22 (0-4)	TRGI cognitions	—
Koenig et al. (20)	CS	373 Veterans w PTSD symptoms	Symptoms only	10 MI symptom categories	45 (1-10)	MISS-M-LF	α = .92 ICC = .91
Zerach and Levi-Belz (60)	CS	191 Israeli combat Veterans	Events and symptoms	Transgressions by self, by others, and betrayal	9 (1-6) 19 (1-4	MIES MIQ-M	— —

### Single or Limited Dimensional Scales

The majority of studies used scales that assessed only one or two dimensions of MI in Veterans and ADM. These studies either a) reported the development of a new scale or b) used previously published scales or subscales that had assessed specific aspects of MI in non-military populations (discussed below by year of publication). We include these scales for background only in this comprehensive review.

Regarding studies reporting the development of a *new scale*, the first was by Henning and Frueh who developed the Combat Guilt Scale (CGS) (30). This measure, which assesses 15 guilt symptoms related to combat experiences, was administered to 40 U.S. Veterans diagnosed with combat-related PTSD. Each symptom was rated as either present or absent, producing a theoretical score ranging from 0 to 15. CGS scores in this study were significantly and positively related to re-experiencing, avoidance, and arousal subscales of the Clinician Administered PTSD Scale and to the total score on the Mississippi Scale for Combat-Related PTSD (with r’s ranging from 0.45 to 0.50).

Stein and colleagues conducted structured clinical interviews with 122 active duty Army personnel, who had experienced traumatic events during their military service (31). Traumatic events were categorized into six groups by two of the authors: life threatening to self, life threatening to others, aftermath of violence, traumatic loss, *moral injury by self* (MI-S), and *moral injury by others* (MI-O). Each category was dichotomized into whether such an event was present (1) or not (0). Relationships were then examined between these categories and various measures assessing emotional reactions to trauma. MI-S was most strongly related to the post-trauma emotions of humiliation, sadness, numbness, PTSD symptoms in the re-experiencing cluster, and guilt symptoms (assessed by the Trauma-Related Guilt Inventory). MI-O was most strongly related to humiliation, anger, and state anxiety. The authors concluded that these findings provided tentative support for the six event categories above. This was one of the first studies to examine combat-related events that might result in MI.

Ritov and colleagues developed a 4-item scale assessing “moral objections” (MO) to commands given by superior officers (33). Participants were 145 reserve combat troops in the Israel Defense Forces. Soldiers were expected to act on these commands (each rated on a 1 to 7 scale from “very little objection” to “very much objection”). Again, those with high MO scores experienced more PTSD symptoms and, interestingly, were more likely to indicate a left lateral preference (despite all being right-handed), possibly suggesting greater right brain activation.

Campbell reported the development of a scale assessing “shame,” called the Military Compass of Shame Scale (M-CoSS) (37). The scale was initially administered to 379 U.S. Navy sailors preparing to deploy to Iraq and Afghanistan, and then to 27 ADM with PTSD undergoing residential treatment. The M-CoSS consists of 10 shame-producing scenarios paired with four maladaptive shame regulation strategies (attack self, attack other, withdrawal, or avoidance). The PTSD sample scored significantly higher on all four subscales of the M-CoSS.

Lancaster administered two 5-item subscales from the 15-item State Shame and Guilt Scale (61), along with an original 7-item measure of transgressive acts (Transgressive Acts Scale; TAS) to 161 Veterans (41). Examples of TAS items included treating civilians more harshly than necessary, perpetrating violence that was out of proportion to the situation, and so forth. The author found a significant direct relationship between the TAS and PTSD symptoms, as well as indirect effects on both PTSD and depressive symptoms through guilt and shame. Psychometrics of the new scale (TAS) were not provided.

Finally, Maguen and colleagues conducted a RCT examining effects of the Impact of Killing (IOK) intervention in 33 combat Veterans with PTSD (42). IOK involves six to eight 60- to 90-min weekly sessions of individual CBT targeting maladaptive thoughts about killing, difficulty with self-forgiveness, spiritual and moral issues, and making amends. Participants were randomized to either IOK (n = 17) or a wait-list control group (n = 16). One of the outcomes examined involved maladaptive beliefs about killing, including beliefs about the justification of killing, wishes not to have killed, and feelings of betrayal from superiors, measured using the 55-item Killing Cognitions Scale (KCS). No psychometrics were provided for the instrument, which the authors indicated was “still being validated.” KCS scores (maladaptive cognitions having to do with killing in war) were significantly reduced in those receiving the IOK intervention compared to those in the wait-listed control group.

Rather than examine MI using a new scale, several studies have used scales or subscales from existing measures originally published and validated in non-military populations or used for purposes other than examining MI. Gray and associates conducted an open trial (without a control group) examining Adaptive Disclosure Therapy (ADT) in 44 active duty Marines (32). One outcome measure was the Posttraumatic Cognitions Inventory (PTCI), a 33-item scale that assesses negative beliefs about the self, negative beliefs about the world, and self-blame (62). No psychometrics were reported in Gray et al.’s sample, although they indicated that the PTCI’s authors had previously found the scale to have high internal consistency and stability (62). While this measure does not assess MI symptoms *per se*, it does assess cognitions that may be driving these symptoms (e.g., “I can’t rely on myself” or “I am inadequate” leading to self-condemnation; “people can’t be trusted” leading to loss of trust; “the event happened because of the way I acted” or “the sort of person I am” leading to guilt or shame, etc.). In the pre-post analysis, ADT significantly decreased PTSD symptoms and depressive symptoms, as well as negative beliefs about the self, world, self-blame, and total PTCI scores.

Bryan (CJ) and colleagues administered the 6-item guilt and 10-item shame subscales of the Personal Feelings Questionnaire (63) to 69 ADM (95% Air Force) seen in military mental health outpatient clinics, examining the relationship between guilt and shame and suicidal ideation or behavior (27). Guilt and shame were both associated with more severe suicidal ideation, findings that were independent of depression and PTSD symptom severity.

Bryan (AO) and colleagues administered the six-item self-forgiveness subscale from the Heartland Forgiveness Scale (64) to 476 ADM and Veterans, examining its relationship to suicidal ideation or attempts (28). We include this study because of the importance of forgiveness (self-forgiveness and forgiveness of others) as a dimension of MI, which has been stressed by experts in this area (11, 14). The results of that report indicated that greater self-forgiveness was inversely related to both suicidal ideation and past suicide attempts in bivariate analyses and in multivariate analyses was inversely related to past suicide attempts, independent of depression and PTSD symptom severity. Bryan et al. concluded that this aspect of MI may help to explain the association between PTSD and suicide risk among military personnel.

Next, Hijazi and colleagues administered the 5-item “wrongdoing” subscale from the 32-item Trauma-Related Guilt Inventory (TRGI) (65) to 167 U.S. Veterans seeking treatment for PTSD, examining its relationship to posttraumatic growth (PTG). (35) Hierarchical regression modeling revealed that non-white ethnicity, greater cognitive flexibility, and *higher scores* on the wrongdoing subscale were associated with greater PTG. While the association between higher scores on the wrongdoing subscale and PTG seems counterintuitive, feelings of wrongdoing may indicate a more sensitive conscience and, with greater cognitive flexibility, drive these individuals to psychologically (and perhaps spiritually) grow from these traumatic experiences, whereas those with less sensitivity to these matters or less cognitive flexibility may be less driven to make the changes necessary for such growth.

In another study assessing guilt and now also shame, Crocker and colleagues examined whether these indicators of MI mediated the relationship between PTSD symptom severity and aggression in 127 U.S. Veterans returning from deployment to the Middle East (36). Guilt was assessed with the 32-item TRGI mentioned earlier, whereas shame was measured using a 24-item subscale of the Internalized Shame Scale (66). Results indicated that while both guilt and shame were associated with higher PTSD severity, only shame mediated the relationship between PTSD severity and aggression.

Yan administered the Combat Experiences (CE) and Aftermath of Battle (AB) subscales from the Deployment Risk and Resilience Inventory (DRRI-1) (67) to 100 U.S. Veterans who served in Operation Enduring Freedom (OEF) and Operation Iraqi Freedom (OIF), examining the relationship between potentially morally injurious events (PMIES) and mental health outcomes. (38) Each of these subscales were assessed with 15 yes or no items. Regression analyses controlling for other predictors revealed that AB scores were inversely related to overall mental health and positively related to depressive symptoms, whereas CE scores were positively related to PTSD symptom severity.

Likewise, Dennis and colleagues examined the relationship between PIES and mental health outcomes in 603 U.S. combat Veterans seeking mental health services for PTSD (39). In this study, investigators administered the Atrocities Exposure Subscale (AES) of the Vietnam Era Stress Inventory (68) along with the four-item global guilt subscale of the TRGI. The AES consists of six items that ask about directly or indirectly being involved in “hurting,” “killing,” or “mutilating bodies” of Vietnamese soldiers or civilians. Structural equation modeling revealed that AES score predicted increased guilt, PTSD severity, hostility, aggression, depression, and suicidal ideation, controlling for combat exposure. Guilt partially mediated the relationship between AES and PTSD severity.

Frankfurt and associates asked questions on commission of transgressive acts (PMIEs) from the Clinician Administered PTSD Scale-IV (69) and feeling guilty from the Mississippi Scale for Combat PTSD (70) to 190 U.S. combat Veterans (40). The purpose was to examine the relationships between responses to these questions and combat exposure, fear, suicidality, and PTSD symptoms using structural equation modeling. Results indicated that guilt again partially mediated the relationship between commission of transgressive acts and both suicidality and PTSD symptoms. Both studies above suggested that MI symptoms may help to explain the negative impact of PMIEs on mental health outcomes, particularly PTSD symptoms.

In one of the few studies of military personnel outside of the U.S., Nazarov and colleagues examined the relationship between PMIEs, PTSD, and depressive symptoms in 4,854 Canadian ADM (reserve ADM deployed to Afghanistan and members of the regular armed forces) (43). The three items asking about PMIE’s were taken from the eight-item deployment experiences (DEX) module of the U.S. Walter Reed Army Institute of Research Combat Experiences Scale (71) adapted for use by the Canadian Department of National Defense. These three items asked whether the respondent had 1) seen ill or injured women or children but was unable to help; 2) had trouble distinguishing combatants and non-combatants; and 3) had been responsible for the death of a Canadian or allied member of the force. Again, PMIEs were associated with both recent PTSD and major depression.

Bryan (CJ) and colleagues administered five three-item subscales of the Differential Emotions Scale-IV (72) (anger, hostility, sorrow, guilt, and shame) and the five-item Unit Social Support Scale from the DRRI-2 (73) (a measure of Unit cohesion) to 930 active duty U.S. National Guard personnel (44). Also given were measures of PTSD, alcohol use, insomnia, and nightmares. The goal was to identify differences between symptoms of MI and PTSD symptoms and then to determine their relationship with suicide risk. Structural equation modeling was used to examine the overlap between MI and PTSD symptoms. Results indicated a five-item factor characterized by nightmares, insomnia, flashbacks, memory loss, and startle reflex (corresponding to the authors’ theorized composition of PTSD) and a six-item factor characterized by low enjoyment, low unit cohesion, anger, shame, guilt, and inward hostility (corresponding to the authors’ theorized composition of MI). An interaction was found between PTSD and MI factors. Suicidal ideation and attempts were associated with PTSD severity, but this was true only in those with high MI scores.

Finally, Currier and colleagues examined Veterans’ preferences for incorporating spirituality into therapies for treating PTSD or major depression (45). Two samples of Veterans were surveyed (499 Veterans from a general population and 624 Veterans who had completed one or more war-zone deployments). Several characteristics were assessed in both samples including severity of PTSD and depressive symptoms. In addition, religious or spiritual struggle (an aspect of MI) was assessed using the Religious and Spiritual Struggles Scale (RSSS). (74) This 26-item measure assesses spiritual struggles related to belief in God, moral issues, religious doubting, meaning and purpose, and interpersonal religious interactions. Researchers found that each of these five religious or spiritual struggle dimensions were positively related to a preference for spiritually integrated treatments (especially in the second sample of Veterans deployed to combat zones).

### Multidimensional Scales

Of the 22 studies, five were designed to assess multiple dimensions of MI in Veterans or ADM. Two of the five scales measure a combination of events and symptoms, and three scales measure MI symptoms alone. We describe each of these measures below.

*Moral Injury Events Scale (MIES)* (12). The nine-item MIES is the first measure designed specifically to assess multiple dimensions of MI in a military population and is the shortest of the five scales. The three dimensions of MI assessed by the MIES are perceived transgressions by self (three items), perceived transgressions by others (three items), and perceived betrayal by others (three items). The MIES assesses both the previous experience of PIES (witnessing acts of commission, perpetrating acts of commission, or perpetrating acts of omission) and symptoms (feelings of distress over acts of commission, omission, or betrayal). The factor structure of the MIES in the original study revealed two MI dimensions (transgressions by self or others and betrayal), which were determined using exploratory factor analysis (EFA) in 533 active duty U.S. Marines and then was replicated using confirmatory factor analysis (CFA) in a second cohort of 506 Marines. However, Bryan and colleagues (22) later reported that the MIES was actually composed of three dimensions (transgressions by self, transgressions by others, and betrayal) in a study of 151 ADM, findings that were replicated in 935 ADM. In the original study (12), the item-to-total correlations on the MIES ranged from 0.55 to 0.79, and the internal reliabilities for each of the two dimensions were high (α = 0.89 for perceived transgressions and α = 0.82 for perceived betrayals). The MIES demonstrated high temporal stability (between 1 and 3 months post-deployment) and discriminant and convergent validity and was significantly and positively related to depressive symptoms (r = 0.40), negative affect (r = 0.29), anxiety (r = 0.28), and PTSD symptoms (r = 0.28), and was inversely associated with social support (r = −0.29) and positive affect (r = −0.15).

The greatest strength and the greatest weakness of the MIES is that it measures both the occurrence of transgressive events and the symptoms associated with those events. Including events that might be the cause of MI symptoms makes it excellent as a screening measure, since it identifies specific events that might be the target of interventions. The inclusion of events, however, means that the MIES might be less useful in intervention studies that seek to assess change in MI symptoms over time, in that the inclusion of MI events in the MIES that cannot change complicates the assessment of MI symptom change in response to treatment.

*Moral Injury Questionnaire-Military Version (MIQ-M)* (34). The 19-item MIQ-M was the second multidimensional scale developed specifically to assess MI in military populations. This measure is made up of a single factor that assesses numerous aspects of MI and also (like the MIES) includes both PMIEs and symptoms that result from those events. *Events* include acts of commission involving betrayal of personal values, acts of revenge or retribution, witnessing or committing moral violations, and witnessing or involvement in the death of innocents or fellow soldiers. *Symptoms* include feelings of betrayal by others or self, guilt over failing to protect others, guilt for surviving when others did not, and feeling changed from experiences had during war. The MIQ-M was initially validated using EFA in 131 Iraq or Afghanistan Veterans attending a community college on the West Coast, and then the factor structure was replicated using CFA in a clinical sample of 82 Veterans receiving residential treatment for PTSD. EFA and CFA of the MIQ-M demonstrated strong fit to the data in both community and clinical samples. Although internal consistency and test-retest reliability were not reported, the MIQ-M was strongly related to combat exposure (r = 0.63), work and social maladjustment (r = 0.42), depressive symptoms (r = 0.39), and PTSD symptoms (r = 0.65), as well as greater risk of suicide in multivariate analyses (B = 0.22, SE = 0.11, p < 0.05), indicating concurrent and incremental validity.

*Moral Injury Symptoms Scale-Military Version (MISS-M)* (23). Two scales that comprehensively measure MI symptoms alone were published online about the same time in late 2017, the 45-item Moral Injury Symptom Scale-Military Version (MISS-M) and the 17-item Expressions of Moral Injury Scale-Military Version (EMIS-M) (26). Not long afterward in 2018, a report on the development of a third scale was published that also measures MI symptoms only, the brief 10-item version of the MISS-M (MISS-M-SF).

The MISS-M-LF (long form) was designed for use in Veterans and ADM with PTSD symptoms. The measure assesses 10 dimensions of MI that capture both the psychological and the spiritual or religious (S/R) symptoms of this construct. Each dimension of the MISS-M-LF was intentionally chosen based on the definitions for MI reported in the literature. Psychological symptoms assessed include guilt (4 items), shame (2 items), betrayal (3 items), moral concerns (3 items), loss of meaning and purpose (4 items), difficulty forgiving (7 items), loss of trust (4 items), and self-condemnation (10 items). S/R symptoms assessed include religious struggles (six items) and loss of religious faith and hope (two items). Items making up the scale were derived primarily from existing scales published in the literature. All items are rated on a scale from 1 to 10 (total score range 45 to 450).

To ensure that items with strong face validity for a particular dimension ended up on the subscale assessing that dimension, EFA and CFA were conducted at the subscale level rather than at the item level. A sample of 427 Veterans and ADM with PTSD symptoms was randomly split into two groups. EFA was performed on an original pool of 54 items in the first half of the sample (n = 214). EFA identified one or two factors per dimension and reduced the total number of items to 45 when only those items with factor loadings ≥ 0.45 were retained. The factor structure for each dimension was then independently verified using CFA in the second half of the sample (n = 213). The final MISS-M-LF had high internal reliability (α = 0.92) and test–retest reliability [intraclass correlation (ICC) = 0.91]. Discriminant validity was demonstrated by relatively weak correlations with S/R measures, community activities, and indicators of physical health; convergent validity was indicated by strong correlations with symptoms of PTSD, anxiety, and depression (r’s ranging from 0.56 to 0.62). The MISS-M-LF is the first multidimensional scale that measures both the psychological and S/R symptoms of MI, and because it measures symptoms alone, the scale can be used for tracking symptom severity in clinical practice and for conducting research that examines treatments for MI in Veterans and ADM that target MI symptoms.

In order to create a shorter measure that might facilitate its use by clinicians and researchers, an abbreviated version of the MISS-M was developed (24). The 10-item MISS-M-SF assesses the same 10 dimensions as the 45-item MISS-M-LF but does so with only one item per dimension (total score ranges from 10 to 100). The sample used for developing the MISS-M-SF was the same used for development of the MISS-M-LF. The highest loading item for each dimension was identified using EFA in the first half of the sample and was verified in the second half of the sample using CFA. The scale had acceptable internal reliability (α = 0.73) and test–retest reliability (ICC = 0.87). The correlation between the short and long versions of the MISS-M-LF was high (r = 0.92). The MISS-M-SF may be easier to use for clinicians and researchers given its brevity and ability to comprehensively assess both the psychological and spiritual symptoms of MI.

*Expressions of Moral Injury Scale-Military Version* (EMIS-M) (26). The 17-item EMIS-M assesses the symptoms of MI across two dimensions: self-directed and other-directed. The self-directed subscale assesses symptoms of guilt, shame, moral concerns, self-condemnation, social withdrawal, and inability to forgive self. The other-directed subscale assesses anger and feelings of betrayal, revenge, and disgust over what others have done. An initial pool of 85 candidate items was reduced down to 45 during a four-stage process by reviewing the literature and consulting with subject experts. EFA was then done in a sample of 286 Veterans to reduce the number of items from 45 down to 17, identifying two factors with strong internal reliability (α = 0.92 for self-directed, α = 0.90 for other-directed). The factor structure was then verified using CFA in a second sample of 624 Veterans (α = 0.94 for self-directed, α = 0.92 for other-directed). Test-retest reliability in the first sample was high for each subscale and the overall scale (ICC = 0.74, 0.80, and 0.80, respectively). Convergent and concurrent validity was demonstrated by strong correlations between the EMIS-M (total score) and PTSD symptoms (r = 0.69 to 0.73), depression (r = 0.58 to 0.65), social support (r = −0.45), and scales assessing other dimensions of MI (r = 0.69 for loss of meaning, r = −0.44 for forgiving others, r = 0.57 for perceived transgressions, and r = 0.62 for perceived betrayals on the MIES). Thus, the EMIS-M is a solid measure of the psychological symptoms of MI and, because it measures symptoms only, can be used by clinicians to follow symptom change with treatment or by researchers to assess the efficacy of interventions that target MI.

### Use of Moral Injury Scales

The MIES is currently the most frequently used multidimensional measure in the literature that assesses PMIEs and MI symptoms, followed by the MIQ-M (**Table 2**). The three multidimensional MI symptom scales (EMIS, MISS-M-LF, and MISS-M-SF) have been published so recently that not enough time has passed yet for investigators to use them. Among the one- or two-dimensional scales used most often are the guilt and shame subscales of the PFQ-2 and the guilt cognitions subscale of the TRGI, although these were not designed specifically for assessing MI in military populations as were the five multidimensional scales above. **Table 3** lists and distinguishes between scales that measure MI events only, MI symptoms only, and both events and symptoms.

**Table 3:** Scales measuring events, symptoms, and events and symptoms.

Events Only	Symptoms Only	Events and Symptoms
Event Categories (31)	Combat Guilt Scale (30)	Moral Injury Events Scale (12)
Vietnam Stress Inventory	Posttraumatic Cognitions Inventory (62)	Moral Injury Questionnaire (34)
(atrocities exposure subscale) (68)	Personal Feelings Questionnaire	Deployment Risk &
Moral Objections Scale (33)	(guilt and shame subscales) (63)	Resilience Inventory (67)
Clinician PTSD Scale-IV	Heartland Forgiveness Scale)	
(transgressive acts subscale) (69)	(self-forgiveness subscale) (64)	
Transgressive Acts Scale (41)	Trauma Related Guilt Inventory (65)	
Combat Experiences Scale (71)	Internalized Shame Scale (66)	
	Military Compass of Shame Scale (37)	
	State Shame and Guilt Scale (61)	
	Killing Cognitions Scale (42)	
	Expressions of Moral Injury Scale (26)	
	Moral Injury Symptoms Scale-LF (23)	
	Moral Injury Symptoms Scale-SF (24)	
	Differential Emotions Scale-IV (72)	
	Religious & Spiritual Struggles Scale (74)	

## Discussion

Moral injury is a term now used widely in clinical discussions and research studies involving Veterans and ADM personnel (11, 75, 76). As MI is discussed more and more in the psychiatric literature, particularly as it applies to those with concurrent PTSD, the comprehensive quantitative measurement of this syndrome will become increasingly important. Studies have shown that the vast majority of Veterans and ADM with PTSD have symptoms of MI from events experienced while serving in the military (20, 21, 76). While MI and PTSD are distinct constructs that frequently occur together, why they are associated (including concerns over definitional overlap) and how MI and PTSD influence each other over time are largely unknown. Longitudinal studies and psychometric studies directly addressing convergent and divergent validity of MI and PTSD measures will be needed to more completely sort this out.

This is the first comprehensive review of MI measures developed specifically for use in current or former military personnel. We described the development of these measures, their psychometric properties, and their relationship to mental health outcomes such as PTSD, anxiety, depression, and suicide risk. These measures assess PMIEs or transgressions, current symptoms of moral conflict over those events, or both events and symptoms. Some scales measure either one or two aspects of MI, whereas others assess multiple dimensions. Because some measures are new (published within the past 12 months), clinicians and researchers have had little opportunity to use them outside of the original validation studies, underscoring the need for future studies.

Nevertheless, it is becoming increasingly clear that MI is a syndrome associated with much distress and comorbidity, making it necessary for clinicians treating Veterans or ADM and for those doing research in these populations to be aware of both earlier and more recent measures. This is particularly important because of the role that MI may play in the pathway that leads from war trauma to the development and maintenance of PTSD (11). The urgency to identify factors that may be driving PTSD is due to the high prevalence of PTSD among Veterans returning home and ADM returning from deployment to combat theaters (15, 16, 77); the devastating impact this disorder has on physical health, quality of life, productivity, and social relationships (78–80); and the resistance to treatment that many patients with PTSD show despite the latest pharmacological and psychological approaches (17, 18). Thus, it is becoming clear that MI is a condition that can no longer be ignored because of both the suffering it causes and the possible negative impact on PTSD.

Further epidemiological research is necessary to determine whether and how MI affects PTSD (and related co-morbidity) over time and how MI is affected by these conditions, all of which requires longitudinal studies have yet to be done. However, given the high prevalence of MI among Veterans and military personnel with PTSD and the frequent lack of recognition by clinicians, it may be important to start now to identify those with significant MI symptoms through screening (81). This requires that clinicians be aware of measures that can assist in case identification, as well as information about treatment options. The development of treatments for MI and establishment of their efficacy likewise requires psychometrically reliable and valid symptom measures that can be targeted by those interventions.

The field, however, is moving fast. Despite knowing relatively little about MI or how it relates to PTSD over time, researchers are now developing and testing interventions to treat some aspects of MI in both Veterans and ADM (82). For example, studies are now taking place or being proposed to examine the efficacy of mainstream and spiritually integrated treatments for MI in former or current military personnel with PTSD symptoms. Mainstream interventions suggested for MI include Cognitive Behavioral Therapy (CBT) (83), Cognitive Processing Therapy (CPT) (84, 85), Prolonged Exposure (PE) (86), Acceptance and Commitment Therapy (ACT) (87), and Adaptive Disclosure Therapy (ADT) (88), many of which have also been used to treat PTSD. Spiritually integrated treatments have also received attention because the moral values that are transgressed in MI are often based on religious beliefs of individuals or of the culture in which they were raised. One such treatment is a group intervention for moral trauma called Building Spiritual Strength (BSS) that is now being delivered in faith community settings (89). Another such treatment is a one-on-one intervention administered by licensed clinicians called Spiritually Integrated CPT (SICPT) that uses the patient’s religious beliefs to process traumatic events and dysfunctional cognitions using a CPT framework (90–92). There is growing evidence of treatment efficacy from pilot interventions directed at specific aspects of MI, such as the guilt from killing in war (42), inner distress from combat using ACT (93), and moral and religious conflicts associated with combat-related trauma (89, 94). Some of these studies are now ongoing (57, 95). Awareness of multidimensional MI symptom scales will facilitate future RCTs examining the efficacy of such interventions.

Thus, many of the MI measures above will be useful for both clinicians working with patients and researchers designing and implementing research studies. However, none of the measures reviewed here was created using a gold standard methodology, such as by starting with representative focus groups to collect a comprehensive list of all possible symptoms, behaviors, affects, and cognitions that might possibly be a result (and component) of MI, and then see what correlates with what, letting the data create the symptom clusters. The EMIS goes a long way in this regard, although possibly not far enough. Without doing such heavy lifting involved in the discovery of symptoms clusters from a much larger pool, researchers cannot be sure that they’ve got the right measure that comprehensively assesses this concept. The development of measures driven solely by statistical grouping, on the other hand, may not be the ideal solution either, since the face validity of items guided by theory should also play some role in determining items for a comprehensive measure of any new construct. That too cannot be ignored.

### Limitations

A number of limitations should be considered when interpreting the results of this review. First, not examined here were MI scales designed to assess symptoms resulting from traumatic experiences occurring outside of the military, such as trauma from assault, rape, or natural or man-made disasters. This may not have always been indicated in the scales. For example, the MIQ-M specifies that MIEs must have occurred in the context of wartime deployment, whereas other measures are not as clear in that instruction. Second, this review was also limited by not including all studies that measured various dimensions of MI (e.g., guilt, shame, difficulty forgiving, self-condemnation, and loss of meaning or trust), particularly those that did not include the term “moral injury” in the title, abstract, or full text of the article (an inclusion criterion for this review). The relative recency of the term “moral injury” likely contributed to missing such studies. However, conducting a review that separately examined each possible dimension of MI (indicated by a wide range of terms) would have gone beyond the scope of this paper. Third, and perhaps most concerning, the present authors developed two of the measures discussed in this review (MISS-M-LF and MISS-M-SF), thus introducing the possibility of bias in study description, particularly since these two measures are recommended for use (see below). In order to address this bias, the authors have described the other three multidimensional measures as comprehensively and accurately as possible, especially the only other “pure” MI symptom measure, the EMIS-M. Despite these efforts, readers should be aware that this bias may have colored our descriptions of these measures. Finally, the scales reviewed here (even those assessing PMIEs) did not always identify the exact circumstances in which Veterans or ADM experienced their trauma, i.e., whether this occurred while fighting in combat, during deployment but not combat, or either before or after returning from deployment, and the specific nature of the trauma (assault, rape, etc.), which clinicians will need to explore beyond simply administering a scale.

### Recommended Scales

As noted earlier, we have included measures in this review that address only certain aspects of MI (e.g., transgressions, guilt, and shame). These measures, in our opinion, are not assessing the complete phenomenon of MI, but rather only certain dimensions of this construct. For this reason, we recommend the use of multidimensional measures that go beyond measuring guilt and shame and are more likely to capture MI as the unique phenomenon that experts in the field now describe (see above). However, given the limitations noted above, these recommendations should be viewed as strictly preliminary rather than instructive.

As always, the scale chosen will depend on the purpose of the clinician or investigator. Multidimensional scales that assess *events* involving transgressions of moral values by self or others and *symptoms* resulting from such transgressions are mostly likely to comprehensively cover the construct of MI. For clinicians wishing to screen current or former military personnel for MI to identify whether this syndrome needs attention, any of the five multidimensional scales described above will serve this purpose. Bear in mind, however, that the questionnaires described here are for screening purposes only and, if positive (i.e., several yes responses to events or symptoms), should be followed by a clinical interview. Unfortunately, none of these measures have established thresholds for the number of clinically meaningful events or symptoms.

The two shortest scales for clinicians are the 9-item MIES (12) and the 10-item MISS-M-SF (24). The advantage of the MIES is that it assesses both events and symptoms, allowing identification of the particular event that may be driving symptoms. The advantage of the MISS-M-SF is that it assesses symptoms only, allowing for the tracking of treatment progress over time, and measures all 10 dimensions of MI, including the religious or spiritual aspects. For researchers wanting to examine the association between MI and mental or physical health outcomes or include MI as a covariate in studies with other objectives, again, any of the five multidimensional scales would be appropriate, depending on how much room is available in the questionnaire for assessing MI. For investigators wishing to conduct intervention studies that target MI in former or current military personnel, only multidimensional “symptom” scales are recommended (since PMIEs experienced in the past are unlikely to change in response to treatment). Multidimensional symptom scales are the EMIS (26), MISS-M-LF (23), and MISS-M-SF (24). To our knowledge, the MISS-M-LF and MISS-M-SF are the only symptom scales now available that assess both the psychological and the religious or spiritual dimensions of MI.

## Conclusion

While the recognition of inner conflict over moral transgressions in former or current military personnel has increased during the past decade, many clinicians and researchers may not know how to measure or treat these injuries. There is growing evidence that MI in Veterans and ADM is associated with adverse mental health states, including PTSD, depression, anxiety, and risk of suicide, and may block the treatment of these conditions unless also addressed. We identified 42 studies in this review that used scales to assess one or more aspects of MI as currently defined. Among those studies, 17 reported the use of scales that assessed only one or two dimensions of MI, while five studies reported the development and psychometric properties of scales assessing multiple dimensions. These measures assess morally injurious events, symptoms that result from the events, or both events and symptoms. Measures that assess both events and consequences are assessing the morally injurious event and the symptoms that the event may cause. Some events may not result in symptoms, whereas some symptoms assessed may not result from the morally injurious event. Therefore, when clinicians are using these scales to screen for MI, a clinical interview will be necessary to clarify which MI symptoms may have followed the acknowledged event, and which MI symptoms may have other causes (possibly prior traumas during youth or adulthood).

In comparing the comprehensiveness, internal consistency, and validity across the five multidimensional measures, the 45-item MISS-M-LF (and shorter 10-item MISS-M-SF) is probably the most comprehensive, assessing 10 dimensions of MI including both psychological and spiritual aspects. With regard to internal consistency and reliability, all five scales have solid psychometric properties, although the 17-item EMIS-M has perhaps the best internal reliability (alphas exceeding 0.92) and test–retest reliability (ICCs in the 0.74 to 0.80 range), as well as strong concurrent validity with PTSD symptoms (r = 0.69–0.73), depression (r = 0.58–0.65), and loss of meaning (r = 0.69), established in large samples. However, except for the 9-item MIES and 19-item MIQ-M, the newer scales have not yet been used in many studies (as noted above), so the performance of these scales (MISS-M-LF, MISS-M-SF, and EMIS-M) in other populations and settings still needs to be demonstrated.

Multidimensional scales that assess both events and symptoms (MIES and MIQ-M) are recommended for clinicians who wish to screen Veterans and military personnel for MI and for researchers who wish to conduct observational studies on this syndrome. Multidimensional scales that assess symptoms only (MISS-M-LF, MISS-M-SF, and EMIS-M), however, are recommended for clinicians and researchers wishing to track change in MI symptoms with treatment. Future longitudinal studies are needed to identify cutoff scores and clinically significant change scores for these measures. Likewise, clinical trials are needed to determine whether treatments directed at MI not only reduce MI symptoms but also impact the many adverse mental health outcomes that have been associated with it.

## Author Contributions

HK is a researcher and psychiatrist at Duke University Medical Center in Durham, NC, USA. He contributed to the literature review and is the main author of this article. NY is a researcher and psychiatrist at the Medical College of Georgia and Charlie Norwood Veterans Administration Medical Center in Augusta, GA. He contributed to the literature review and the writing and editing of this paper. MP is a researcher and psychologist at the University of Maryland. She contributed to the literature review and the writing and editing of this paper. In addition, HK, NY, and MP all made important intellectual contributions to this article.

## Funding

This research was not supported by a grant from a funding agency in the commercial, public, or not-for-profit sectors. The study received no funding from any outside funding bodies. The study authors’ time was covered by their individual departments.

## Conflict of Interest Statement

The authors declare that the research was conducted in the absence of any commercial or financial relationships that could be construed as a potential conflict of interest.
